# Increased usage of doxycycline for young children with Lyme disease

**DOI:** 10.3389/frabi.2024.1388039

**Published:** 2024-05-21

**Authors:** Amy D. Thompson, Desiree N. Neville, Laura L. Chapman, Fran Balamuth, Meagan M. Ladell, Anupam B. Kharbanda, Rachael Aresco, Lise E. Nigrovic

**Affiliations:** ^1^Division of Emergency Medicine, Nemours Children’s Hospital and Sidney Kimmel Medical College of Thomas Jefferson University, Wilmington, DE, United States; ^2^Division of Emergency Medicine, Children’s Hospital of Pittsburgh and University of Pittsburgh School of Medicine, Pittsburgh, PA, United States; ^3^Division of Pediatric Emergency Medicine, Rhode Island Hospital and Warren Alpert Medical School of Brown University, Providence, RI, United States; ^4^Department of Pediatrics, Children’s Hospital of Philadelphia and University of Pennsylvania, Philadelphia, PA, United States; ^5^Department of Pediatric Emergency Medicine, Children’s Hospital of Wisconsin and Medical College of Wisconsin, Milwaukee, WI, United States; ^6^Department of Emergency Medicine, Children’s Minnesota, Minneapolis, MN, United States; ^7^Division of Emergency Medicine, Boston Children's Hospital and Harvard Medical School, Boston, MA, United States

**Keywords:** Lyme disease, children, doxycycline, dental, *Borrelia burgdorferi*

## Abstract

**Background:**

The 2018 Infectious Disease Committee of the American Academy of Pediatrics stated that up to 3 weeks or less of doxycycline is safe in children of all ages. Our goal was to examine trends in doxycycline treatment for children with Lyme disease.

**Methods:**

We assembled a prospective cohort of children aged 1 to 21 years with Lyme disease who presented to one of eight participating Pedi Lyme Net centers between 2015 and 2023. We defined a Lyme disease case with an erythema migrans (EM) lesion or positive two-tier Lyme disease serology categorized by stage: early-localized (single EM lesion), early-disseminated (multiple EM lesions, cranial neuropathy, meningitis, and carditis), and late (arthritis). We compared doxycycline treatment by age and disease stage and used logistic regression to examine treatment trends.

**Results:**

Of the 1,154 children with Lyme disease, 94 (8.1%) had early-localized, 449 (38.9%) had early-disseminated, and 611 (53.0%) had late disease. Doxycycline treatment was more common for older children (83.3% ≥ 8 years vs. 47.1% < 8 years; *p* < 0.001) and with early-disseminated disease (77.2% early-disseminated vs. 52.1% early-localized or 62.1% late; *p* < 0.001). For children under 8 years, doxycycline use increased over the study period (6.9% 2015 to 67.9% 2023; odds ratio by year, 1.45; 95% confidence interval, 1.34–1.58).

**Conclusion:**

Young children with Lyme disease are frequently treated with doxycycline. Prospective studies are needed to confirm the safety and efficacy of doxycycline in children younger than 8 years, especially for those receiving courses longer than 3 weeks.

## Introduction

For many years, tetracycline use in children under 8 years of age was limited because of concerns about tooth staining and enamel hypoplasia (Stultz and Eiland, 2019). A recent review of the available evidence suggests that the risks from the newer tetracycline antibiotics (e.g., doxycycline) are very low (Ravindra et al., 2023). In 2018, the Infectious Disease Committee of the American Academy of Pediatrics stated that short courses (up to 3 weeks) of oral doxycycline are safe in patients of all ages (Kimberlin et al., 2018). Then, in 2020, the Infectious Disease Society of America (IDSA), American College of Rheumatology (ACR), and American Academy of Neurology (AAN) suggested that doxycycline be considered a first-line treatment option for Lyme disease-associated meningitis and cranial neuropathy (Lantos et al., 2021). The impact of these guidelines on antibiotic prescribing for Lyme disease, especially for the youngest children, has not been evaluated.

To this end, we assembled a multi-center cohort of children with Lyme disease presenting to one of the eight participating Pedi Lyme Net centers. We identified children diagnosed with confirmed Lyme disease and examined trends in doxycycline treatment over time by patient age and disease stage.

## Methods

### Study design/setting

We performed a prospective cohort study at eight pediatric emergency departments (EDs), located in Lyme disease endemic areas, participating in the Pedi Lyme Net clinical research network (Nigrovic et al., 2020). The study staff approached children undergoing evaluation for potential Lyme disease to obtain informed consent for study participation. The study protocol was approved by the institutional review board at each participating center with permission for data sharing.

### Participants

The parent study enrolled children aged 1 to 21 years of age undergoing evaluation for Lyme disease between June 1, 2015 and December 31, 2023. We limited this sub-study to those children with confirmed Lyme disease and available documentation of antibiotic treatment.

### Data collection

We obtained patient demographics and clinical history from providers and patients at the time of enrollment. Approximately 1 month after enrollment, we reviewed the medical record to abstract results of diagnostic testing as well as the antibiotics prescribed with duration (in days). When antibiotic treatment prescribed and/or duration was not available from the medical record, the study staff performed phone follow-up to clarify.

### Lyme disease case definition

We defined a case of Lyme disease by either provider-diagnosed erythema migrans (EM) lesion or a positive two-tier serology within 30 days of enrollment in a child with symptoms compatible with acute Lyme disease (Mead et al., 2019). Although specific Lyme disease assays varied by study site (Maulden et al., 2020), we utilized the results available to the medical providers making antibiotic treatment decisions. We categorized all Lyme disease cases as one of the following stages after review of the prospectively recorded clinical history and physical examination: early-localized (single EM lesion), early-disseminated (multiple EM lesions, cranial neuropathy, meningitis, carditis), and late (arthritis) (Lantos et al., 2021).

### Primary outcome

Our primary outcome was any treatment with doxycycline. We included all antibiotics prescribed within 30 days of enrollment, documented by medical record review or phone follow-up.

### Statistical analysis

Continuous data were reported using medians and interquartile ranges and categorical data using proportions with associated 95% confidence intervals (CI). First, we compared children treated with any doxycycline to children not receiving doxycycline. Second, we compared the proportion of children treated with doxycycline overall by age (<8 years of age vs. ≥ 8years of age) and by disease stage (early-localized vs. early-disseminated vs. late stage) using chi-square test. Using binary logistic regression, we examined trends in doxycycline use overall and by age as well as disease stage over the study period.

All analyses were performed using SPSS version 29.0.0 (IBM Corp; Armonk, NY, USA).

## Results

Of the 1,154 children with Lyme disease, 1,127 (97.7%) patients had their antibiotic treatment documented. Of these, the median patient age was 8 years [interquartile range (IQR), 6–12 years], and 727 (63.0%) were male patients. The majority of children presented during the peak Lyme disease season between June and October (771, 66.8%). Overall, 81 (7.0%) had an EM lesion alone, 967 (83.8%) had positive two-tier Lyme disease serology alone, and 106 (9.2%) had both. Overall, 94 (8.1%) had early-localized, 449 (38.9%) had early-disseminated, and 611 (53.0%) had late Lyme disease.

Of the 1,127 with antibiotic treatment documented, 756 (67.1%) received doxycycline. Overall, 683 (90.3%) received doxycycline at the index encounter, and 73 (9.7%) were switched to doxycycline after another initial antibiotic. The median duration of doxycycline treatment was 21 days (IQR, 14–28 days). Of the 353 children who received more than 3 weeks of doxycycline, 124 (35.1%) were under 8 years of age.

Next, we compared children treated with doxycycline to those who did not receive doxycycline (**Table 1**). Children treated with doxycycline were older and more likely to have early-disseminated disease.

**Table 1:** Comparison between children treated with doxycycline vs. other antibiotics.

	Doxycycline *N* = 756 *N* (%)	Other antibiotics *N* = 371 *N* (%)
Demographics		
Age (years)^a^	9 (7, 13)	6 (4, 8)
Age group <8 years ≥8 years	244 (32.3%) 512 (67.7%)	268 (72.2%) 103 (27.8%)
Male gender	483 (63.9%)	230 (62.0%)
Peak Lyme season^b^	508 (67.2%)	248 (66.8%)
Clinical history		
Lyme disease stage Early-localized Early-disseminated Late	49 (6.5%) 335 (44.3%) 372 (49.2%)	45 (12.1%) 99 (26.7%) 227 (61.2%)

a Median (Interquartile range).

b Presentation between June and October.

**Figure 1:**
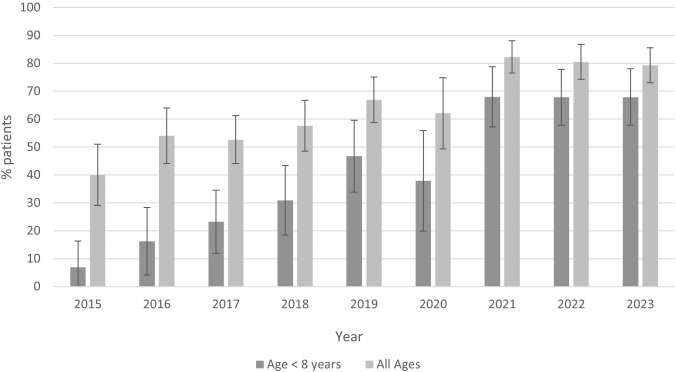
Proportion of children with Lyme disease treated with doxycycline overall and for children <8 years of age (2015–2023).

Overall, the usage of doxycycline increased over the 9-year study period (**Figure 1**). Between 2015 and 2023, doxycycline use increased from 40.0% to 79.3% overall [odds ratio (OR) by year 1.26, 95% confidence interval 1.20–1.33], from 6.9% to 67.9% for children under 8 years (OR by year 1.45, 95% CI 1.34–1.58), and from 42.1% to 84.7% for children with early-disseminated disease (OR 1.30, 95% CI 1.18–1.42).

## Discussion

Consistent with the new safety recommendations (Kimberlin et al., 2018) and Lyme disease treatment guidelines (Lantos et al., 2021), two-thirds of children with confirmed Lyme disease were treated with doxycycline in our multi-center prospective cohort study. Between 2015 and 2023, doxycycline utilization increased substantially, especially for children younger than 8 years of age and those with early-disseminated disease.

Tetracyclines, including doxycycline, are used to treat a wide range of infections and have a low risk of associated serious adverse events (Smith and Leyden, 2005; Agwuh and MacGowan, 2006). However, tetracycline has a high binding affinity for calcium, which can cause enamel hypoplasia and teeth staining (Sánchez et al., 2004). The risk is highest when the exposure occurs before the enamel of the permanent tooth has calcified (under 8 years), especially with higher doses and longer duration of therapy (Conchie et al., 1970; Grossman et al., 1971). Given the risk for dental staining, tetracyclines have not been recommended for children under 8 years of age. Doxycycline, a newer tetracycline with a lower affinity for calcium binding (Forti and Benincori, 1969), does not appear to have the same dental effects at the currently recommended dosages (Cross et al., 2016; Stultz and Eiland, 2019; Ravindra et al., 2023).

While intravenous ceftriaxone has long been the recommended treatment for Lyme neuroborreliosis (Wormser et al., 2006), parenteral antibiotic therapy has been associated with a high rate of complications from both the antibiotic and the commonly required peripherally inserted central catheter line (Thompson et al., 2012). With its high oral availability and known activity against *Borrelia burgdorferi*, doxycycline provides an attractive treatment alternative (Saivin and Houin, 1988; Wormser and Halperin, 2008). Several European clinical trials in adults (Borg et al., 2005; Ljøstad et al., 2008; Kortela et al., 2021; Arnason and Skogman, 2022) and a retrospective study in children (Lopez et al., 2019) reported good outcomes for patients with Lyme meningitis treated with doxycycline. In 2020, the Infectious Disease Society of America, American Academy of Neurology, and American College of Rheumatology recommended either oral doxycycline or parenteral ceftriaxone as a first-line treatment for Lyme meningitis (Lantos et al., 2021). An ongoing prospective study is comparing the efficacy of doxycycline to ceftriaxone for the treatment of pediatric Lyme meningitis on short- and long-term clinical outcomes (Nigrovic et al., 2023a).

Consistent with previous studies, we observed an increase in doxycycline treatment for children with Lyme disease that began before the publication of the recent clinical guidelines. For children with Lyme meningitis diagnosed at one of the 24 pediatric centers located in endemic areas that contributed data to Pediatric Health Information System (PHIS), ceftriaxone utilization declined from 48% in 2015 to 9% in 2020 (Roelf et al., 2021). In a single-center retrospective study of 32 children under 8 years of age with Lyme disease treated with doxycycline, none had treatment failure documented in the medical record (Brown et al., 2023). Although two caregivers reported dental staining on a follow-up telephone survey, this was not based on an in-person dental examination. The current guidelines suggest limiting doxycycline to 3 weeks or less. However, a substantial minority of children under 8 years of age with Lyme disease were treated with a longer course. Although no adverse events were reported, further studies are needed to evaluate the safety of doxycycline courses longer than 3 weeks for young children.

The black-legged tick that transmits Lyme disease can also carry other bacteria, viruses, or parasites that can cause human disease. Doxycycline effectively treats two of the most common bacterial co-infecting pathogens, *Anaplasma* and *Erlichia* (Krause et al., 2021). Therefore, clinicians may select doxycycline to cover *Borrelia* as well as other bacterial coinfections. Using a multiplex high-definition polymerase chain reaction assay (HDPCR), we previously found that only a minority of children with Lyme disease had a tick-borne coinfection, suggesting that the primary reason doxycycline was prescribed was for Lyme disease treatment (Nigrovic et al., 2023b). The optimal approach to the diagnosis and treatment of children with potential tick-borne co-infections requires further study.

Our study has several limitations. First, enrollment was limited to the times when the study staff were available. However, the study patients had similar clinical characteristics to eligible children who were not enrolled. Second, as we did not collect the reason that a specific Lyme disease treatment was prescribed, we cannot take into account antibiotic allergies or concerns for a tick-borne co-infection. Lastly, we only performed a short-term clinical follow-up by review of medical record and phone follow-up and may have failed to identify all treatment complications, including dental staining. Further studies are needed to better understand the potential adverse effects of longer courses of doxycycline for Lyme disease treatment, especially for the youngest children.

## Conclusions

In this eight-center multicenter study, children with Lyme disease were commonly prescribed doxycycline with increasing utilization over the study period (2015–2023), especially for children under 8 years of age and those with early-disseminated Lyme disease. Prospective studies are needed to confirm the safety and efficacy of doxycycline in children, especially for children younger than 8 years receiving it for longer than 3 weeks.

## Data availability statement

The raw data supporting the conclusions of this article will be made available by the authors, without undue reservation.

## Ethics statement

The studies involving humans were approved by Institutional Review Board at each participating center. The studies were conducted in accordance with the local legislation and institutional requirements. Written informed consent for participation in this study was provided by the participants’ legal guardian or by the patient if ≥ 18 years.

## Author contributions

AT: Writing – review & editing, Writing – original draft, Visualization, Validation, Project administration, Methodology, Investigation, Formal analysis, Data curation, Conceptualization. DN: Writing – review & editing, Project administration, Investigation, Data curation. LC: Writing – review & editing, Project administration, Investigation, Data curation. FB: Writing – review & editing, Project administration, Investigation, Data curation. ML: Writing – review & editing, Project administration, Investigation, Data curation. AK: Writing – review & editing, Project administration, Investigation, Data curation. RA: Writing – review & editing, Project administration, Data curation. LN: Writing – review & editing, Writing – original draft, Visualization, Validation, Supervision, Resources, Project administration, Methodology, Investigation, Funding acquisition, Formal analysis, Data curation, Conceptualization.
